# Three-dimensional dynamic homogenous modeling: The biomechanical influences of leg tissue stiffness on pressure performance of compression biomedical therapeutic textiles

**DOI:** 10.3389/fbioe.2024.1418047

**Published:** 2024-07-12

**Authors:** Yu Shi, Chongyang Ye, Rong Liu

**Affiliations:** ^1^ School of Fashion and Textiles, The Hong Kong Polytechnic University, Hong Kong Special Administrative Region (SAR), Kowloon, Hong Kong SAR, China; ^2^ Laboratory for Artificial Intelligence in Design, Hong Kong Science Park, Kowloon, Hong Kong SAR, China

**Keywords:** biomechanical analysis, numerical simulation, tissue stiffness characteristics, medical compression textiles, pressure supply

## Abstract

Patient compliance and therapeutic precision of compression textiles (CTs) are frequently limited by the inaccurate pressure distributions along biological bodies in physical-based compression therapy. Therefore, the biomechanical influences of physiological tissue material characteristics of lower extremities on compression generations of CTs need to be explored systematically to improve pressure management efficacy. In this study, we developed three-dimensional (3D) homogenous finite element (FE) CT-leg systems to qualitatively compare the pressure diversities along lower limbs with different biomaterial tissue properties under each external compression level. Simultaneously, through the obtained leg circumferential displacement, a contact analysis model was applied to quantitatively explore the impact mechanisms of soft leg indentations on the pressure performance of CTs. Based on the experimental validation study, the proposed FE systems could be efficiently utilized for compression performance prediction (error ratio: 7.45%). Through the biomechanical simulation and theoretical calculations, the tissue stiffness characteristics of applied bodies showed significant correlations (*p* < 0.05) with the body circumferential displacements but no correlations (*p* > 0.05) with pressure delivery differences of CTs. This study facilitates the pressure fit design principle and leg mannequin material selection guidance for the development and experimental assessment of CTs. It also provides effective simulation methods for pressure prediction and property parametric optimization of compression materials.

## 1 Introduction

Functional textile-based compression interventions are generally considered as acceptable and effective therapeutic modalities for venous ulceration, chronic venous insufficiency, and deep vein thrombosis (Liu et al., 2017; Kankariya et al., 2021; Kankariya, 2022). By applying controllable fabric tensions during wearing for an extended time, elastic compression textiles (CTs) positively generate external pressure dosages along the required bodies for compression therapy (Barhoumi et al., 2020). The pressure generation/performance of CTs indicate the generated pressure magnitudes between the interfaces of body skin surface and CT fabrics. Through population-based studies, although the prevalence of venous diseases has increased approximately from 8.9% to 16.5% (Gong et al., 2020; Kim et al., 2021), patient compliance is still only 25.6% (Ziaja et al., 2011), which is limited by the inaccurate pressure distributions and discomfort when wearing CTs.

**FIGURE 1:**
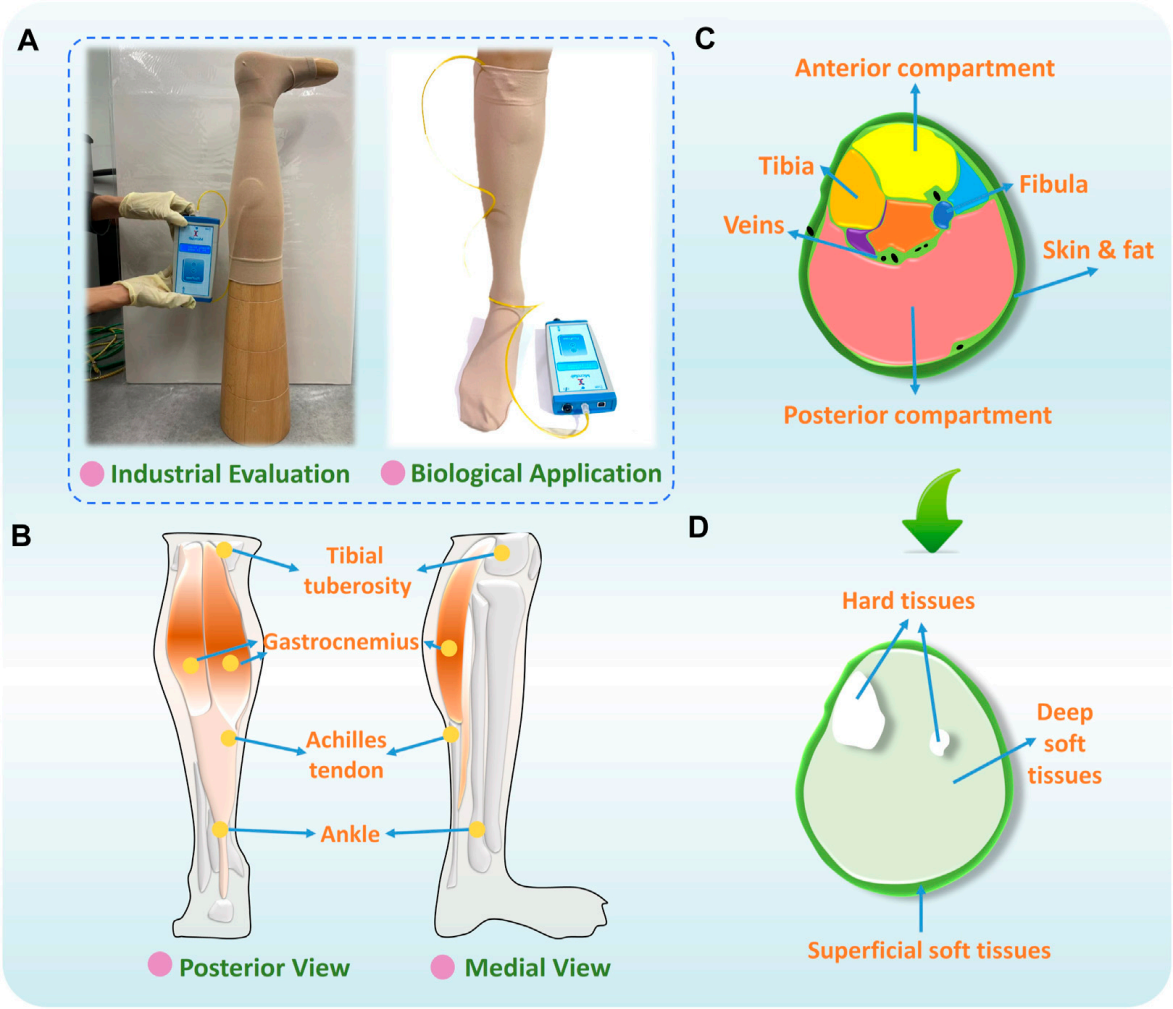
**(A)** Quality evaluation and bio-application of CTs, **(B)** physiological structure, **(C)** sectional segmentation, and **(D)** main compositions of human lower extremity.

The pressure diversities are the pressure value differences generated by identical CTs among various applied bodies. In the production process, the fabricated commercial CTs are necessarily measured by using standard-sized leg mannequins (i.e., wooden leg models) for quality control estimations (Figure 1A). Therefore, insufficient compression deliveries are frequently generated, which are caused by the cross-sectional shape profile and material property discrepancies between the applied leg models and biological bodies (Li et al., 2020). Due to individual physiological diversity and anatomic structural differences (Liu et al., 2013), including morphological irregularities and heterogeneous tissue characteristics (Ghosh et al., 2008; Liu et al., 2018a), the medical efficiency and user adherence of readymade CTs have been limited by inappropriate pressure generation in practical bio-applications. In compression therapy, the interfacial pressure distributions determine the clinical effectiveness of CT materials (Kankam et al., 2018). Thus, the scientific biomechanical analysis of the impact of lower limb individual diversity on compression delivery could improve the pressure fitness and therapeutic precision of CTs.

For lower extremity morphological variations, research workers (Liu et al., 2018b) have explored the impacting mechanisms of body sectional irregularities on functional compression performances of CTs. They found that the cross-sectional irregular applied leg patterns led to uneven pressure magnitudes caused by the non-constant fabric stretching strains. For lower limb material tissues, based on the leg’s physiological structure (Figure 1B) and sectional segmentation (Figure 1C), three components with varying composition have been identified, including the superficial tissues (i.e., adipose tissues, skin, and veins), deep (i.e., muscles, tendons, and veins) soft tissues (STs), and hard tissues (i.e., tibia and fibula) (Figure 1D) (Cieślak et al., 2016). Therefore, except for the rigid support (i.e., bones), to replace traditional rigid mannequin materials and improve the accuracy of experimental pressure estimation, in previous studies, research workers have selected alternative ST materials, such as silicone and flexible polyurethane foam (Yu et al., 2004; Frauziols et al., 2017; Li et al., 2020). Using these, research workers have developed substitutable leg models for the design and interfacial pressure testing of CTs. Nevertheless, to date, there is a lack of research fundamentally exploring the effects of biomaterial mechanical stiffness of lower limbs on pressure distributions of CTs. The design guidance and leg mannequin selection criteria need to be established for the biodevelopment and pressure assessment of compression functional fabrics.

Based on non-linear mechanical behaviors, the applied strain energy functions of biological STs can be commonly defined by neo-Hookean, Mooney–Rivlin, first-order Ogden, or Fung material models, *etc*. (Heydon, 2011; Parker, 2016). For digitalized property investigations, the ST constitutive parameters were measured by direct identification experiments through ultrasound shear wave elastography (Ranger et al., 2023; Frauziols et al., 2013; Mo et al., 2022; Affagard et al., 2014). Furthermore, the computational finite element (FE) simulations and inverse methodologies were also applied to numerically quantify leg ST biomaterial properties. Among them, the subjects’ legs were reconstructed through reverse engineering technologies by employing the magnetic resonance imaging or computed tomography scanning (Avril et al., 2011; Dubuis et al., 2012; Lu et al., 2021). Therefore, through the aforementioned determination approaches and obtained mechanical parameters of STs, the stress and strain distributions, interfacial pressure mapping, and hemodynamic response could be visually simulated through FE biomechanical approaches (Liu et al., 2019; Nemati and Shojaei, 2019; Han et al., 2021). For instance, based on the imaging-based reconstruction of the subjects’ lower limbs, Ye and Liu. (2020) constructed FE fluid–solid complex interaction systems to analyze the biomechanical properties of veins and STs under CT external compressions. Dubuis et al. (2012), Avril et al. (2011), and Rohan et al. (2015) also examined the pressure transmissions within the ST by simulated FE models to improve the understanding of working mechanisms of CTs. However, the previously proposed FE models were established for patient-specific mechanical analysis with their individual biomaterial tissue characteristics. Limited studies have investigated the pressure performance diversities caused by lower limb stiffness variations through parametric comparisons.

Based on characterization studies, ST stiffness values possibly vary due not only to the studied anatomical locations and muscle measured states but also to individual characteristics, such as gender (Morse, 2011), aging (Ochi et al., 2010), disease progression, occupation (Hobara et al., 2010), and rehabilitation status (Le et al., 2017). Additionally, research workers (Sangpradit et al., 2011; Marinopoulos et al., 2020) have explored the interactions between the external forces and positive exerting devices among soft bodies with varying material characteristics. Nevertheless, the garment-based pressure diversities caused by the mechanical ST properties of bio-legs still remain controversial. Thus, the qualitative influencing mechanisms on, and quantitative relationships of various biomaterial parameters to, pressure generation diversities of compression fabrics need to be explored systematically, to facilitate the material design, pressure dosage selection, and compression prediction of CTs.

Therefore, the main objective of this study was to systematically investigate the biomechanical influences of tissue stiffness of lower extremities on compression generations of CTs for the improvement of pressure management efficacy. The independent leg stiffness influences on pressure performances of CTs were systematically investigated through FE CT-leg simulation modeling, theoretical analysis, and experimental validation studies. For our main research contribution, the compared results could facilitate the scientific biodesign of pressure dosages for CTs and promote model material selection for pressure experimental evaluations. The constructed 3D homogenous FE CT-leg systems also achieve effective visualized assessment and pressure prediction for user-oriented applications.

## 2 Materials and methods

**FIGURE 2:**
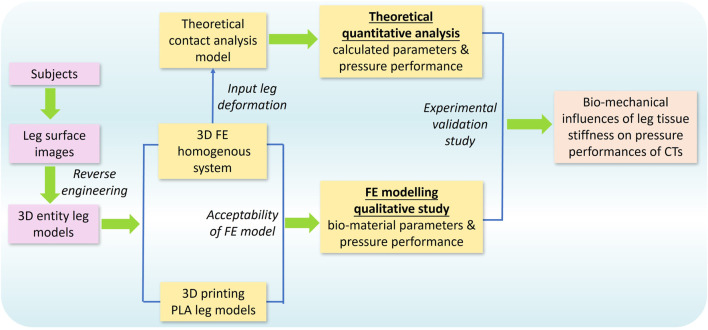
Framework of the proposed study.

To investigate the biomechanical influences of lower limb ST stiffness on pressure performance of CTs, this study adopted FE modeling, theoretical analysis, and experimental validation, respectively. As illustrated in Figure 2, the 3D FE homogenous modeling system was constructed through the reconstructed entity legs established by 3D body scanning and reverse engineering technologies. The acceptability of proposed FE models was examined by 3D printing legs with controlled lower limb morphological shapes and identical material characteristics. Then, the pressure performance diversities between legs with varied tissue stiffness were qualitatively compared through the FE CT-leg systems by inputting varying biomaterial parameters. Simultaneously, the theoretical contact analysis model was constructed to quantitatively explore the impacting mechanisms of tissue stiffness on pressure performances of CTs by inputting leg circumferential deformation data. Finally, the experimental validations were adopted to validate the applicability and accuracy of the outcomes and findings of the present study.

### 2.1 Subject information

Three subjects (code: S1, S2, and S3) with various genders, ages, and leg shape profiles participated voluntarily for lower extremity modeling constructions and experimental investigations. The basic individual information and body mass index (BMI) of each subject are as follows: i) S1: male, age: 32 years old, height: 1.80 m, BMI: 17.7 kg/m^2^; ii) S2: female, age: 50 years old, height: 1.57 m, BMI: 25.6 kg/m^2^; and iii) S3: male, age: 61 years old, height: 1.60 m, BMI: 22.2 kg/m^2^. The study protocol was approved by the Human Subjects Ethics Sub-committee of The Hong Kong Polytechnic University.

### 2.2 Preparation and physical-mechanical properties of CTs

**FIGURE 3:**
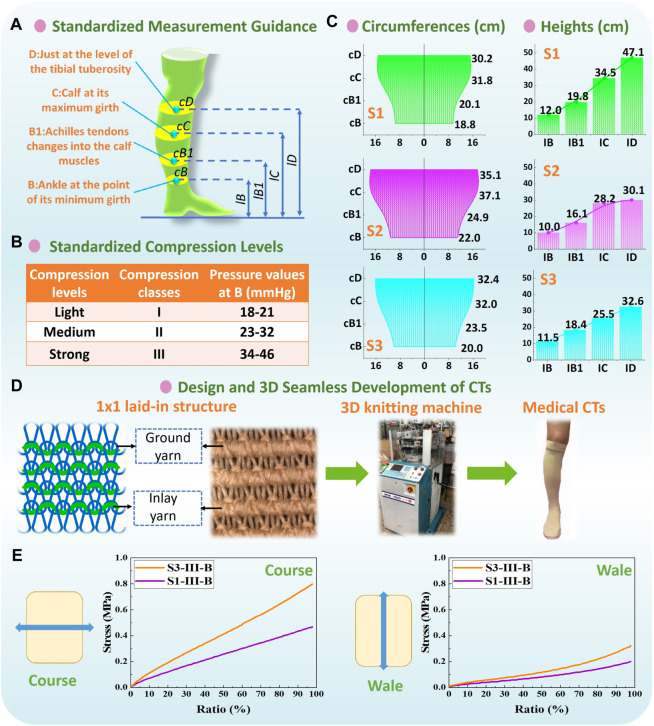
**(A)** Measurement guidance and **(B)** compression classification by Germany RAL-GZ 387 standard. **(C)** Circumferences and heights of each recruited subject legs, and **(D)** applied 1 × 1 laid-in knitting loop pattern and 3D seamless knitting fabrication. **(E)** Typical stress–strain curves of CT samples from stretching tests.

In practical clinical treatments, pressure magnitudes exerted by CTs are classified as different compression levels (Figure 3A) through the standard of Germany RAL-GZ 387 (Medical Compression Hosiery Quality Assurance) by varying textile material stiffness. Thus, according to the measurement guidance (Figure 3B) and determined subject body dimensions (Figure 3C), different compression knitted fabrics were designed to achieve the standardized pressure magnitudes for the light (class: I) and strong (class: III) levels of compression generation. As shown in Figure 3D, Lycra-based elastic yarn materials with various linear densities were adopted as the ground and inlay yarn components according to the designed 1 × 1 laid-in knitted loop pattern. By adjusting the knitting yarn combinations and machinery parameters, tubular CTs with diverse physical and mechanical properties were prepared using the LONATI LA-45 ME 3D seamless knitting machine (Francesco Lonati, Brescia, Italy).

For experimental physical tests, the fabric circumferential radius (*R*_*F*_), longitudinal lengths (*L*_*F*_), fabric thickness (*h*), and mass densities (*MD*) of CTs were obtained according to the standards of ASTM D3774, ASTM D1777, and ASTM D 3776/D 3776M-09a, respectively. For mechanical tensile behaviors, the elastic Young’s modulus along the course (*E*_*F*_) and wale (*E*_*Fy*_) stretching directions and Poisson’s ratio (*v*_*F*_) were tested by utilizing the Instron 4,411 universal tension tester (Norwood, MA, USA) referred to the ASTM D2256 standard. For stretching tests, to facilitate the appropriate sample size, the knitted samples (width: 50 mm and length: 75 mm) were prepared by the same yarn-machinery settings with the corresponding CT fabrics. The samples were fixed by the two tester clamps and thus were stretched from the initial tension-free state (0%) to the maximum final stretch ratio (100%) at a constant extension velocity of 300 mm/min. Young’s moduli values along various loading directions were calculated by *E*_*F-Fy*_ = *F*_*T*_/*bhε* (where *F*_*T*_ is the fabric tension force, *h* and *b* are the fabric thickness and length, respectively, and *ε* is the fabric extension stretched by the corresponding tension force). Thus, through the measured fabric *h* and *b* data, *F*_*T*_ values under the corresponding fabric strain state (*ε*; 0%–100%) were recorded by the Instron device, and then *E*_*F-Fy*_ could be obtained by the averaged calculated results with different stretching strains. The typical stress–strain curves of CT samples are shown in Figure 3E. Fabric shear modulus (*G*_*F*_) was obtained using the Kawabata (KES-FB3) pure shear testing assessment system. The applied CTs and corresponding measured fabric properties are listed in Table 1.

**TABLE 1:** Measured fabric properties of CT knitted samples.

Subject	Class	Leg part	Physical property				Mechanical property			
			*R*_*F*_ (cm)	*L*_*F*_ (cm)	*h* (mm)	*MD* (kg/m^3^)	*E*_*F*_ (MPa)	*E*_*Fy*_ (MPa)	*v*_*F*_	*G*_*F*_ (MPa)
S1	I	B	2.52	6.0	0.66	479.3	0.35	0.16	0.21	0.14
		B1	2.79	6.5	0.67	468.2	0.33	0.15	0.21	0.14
		C	3.57	7.5	0.68	481.3	0.37	0.21	0.20	0.17
		D	3.50	7.0	0.67	480.6	0.38	0.20	0.20	0.16
	III	B	2.42	6.0	0.62	503.6	0.42	0.14	0.22	0.17
		B1	2.79	6.5	0.64	481.5	0.41	0.17	0.23	0.17
		C	3.25	7.5	0.66	479.3	0.35	0.16	0.21	0.14
		D	3.22	7.0	0.68	468.2	0.33	0.15	0.21	0.14
S2	I	B	2.84	5.5	0.69	484.7	0.38	0.21	0.21	0.14
		B1	3.22	6.0	0.67	468.2	0.37	0.15	0.21	0.15
		C	3.57	6.5	0.67	486.0	0.38	0.21	0.20	0.15
		D	3.50	6.0	0.66	485.4	0.37	0.20	0.19	0.14
	III	B	2.83	5.5	0.62	503.7	0.45	0.19	0.23	0.18
		B1	2.87	6.0	0.67	484.9	0.38	0.22	0.20	0.15
		C	3.22	6.5	0.68	468.2	0.34	0.15	0.20	0.14
		D	3.25	6.0	0.66	480.1	0.33	0.16	0.20	0.14
S3	I	B	2.79	5.5	0.68	468.2	0.33	0.15	0.21	0.14
		B1	3.22	6.0	0.68	484.4	0.38	0.21	0.21	0.15
		C	3.50	7.0	0.67	487.3	0.38	0.21	0.20	0.16
		D	3.57	6.5	0.67	486.3	0.38	0.21	0.21	0.15
	III	B	2.80	5.5	0.64	532.7	0.76	0.27	0.25	0.30
		B1	2.83	6.0	0.62	503.6	0.42	0.18	0.22	0.17
		C	3.25	7.0	0.66	479.3	0.35	0.16	0.21	0.14
		D	3.22	6.5	0.68	468.2	0.33	0.15	0.21	0.14

### 2.3 Development of 3D printing rigid leg models

**FIGURE 4:**
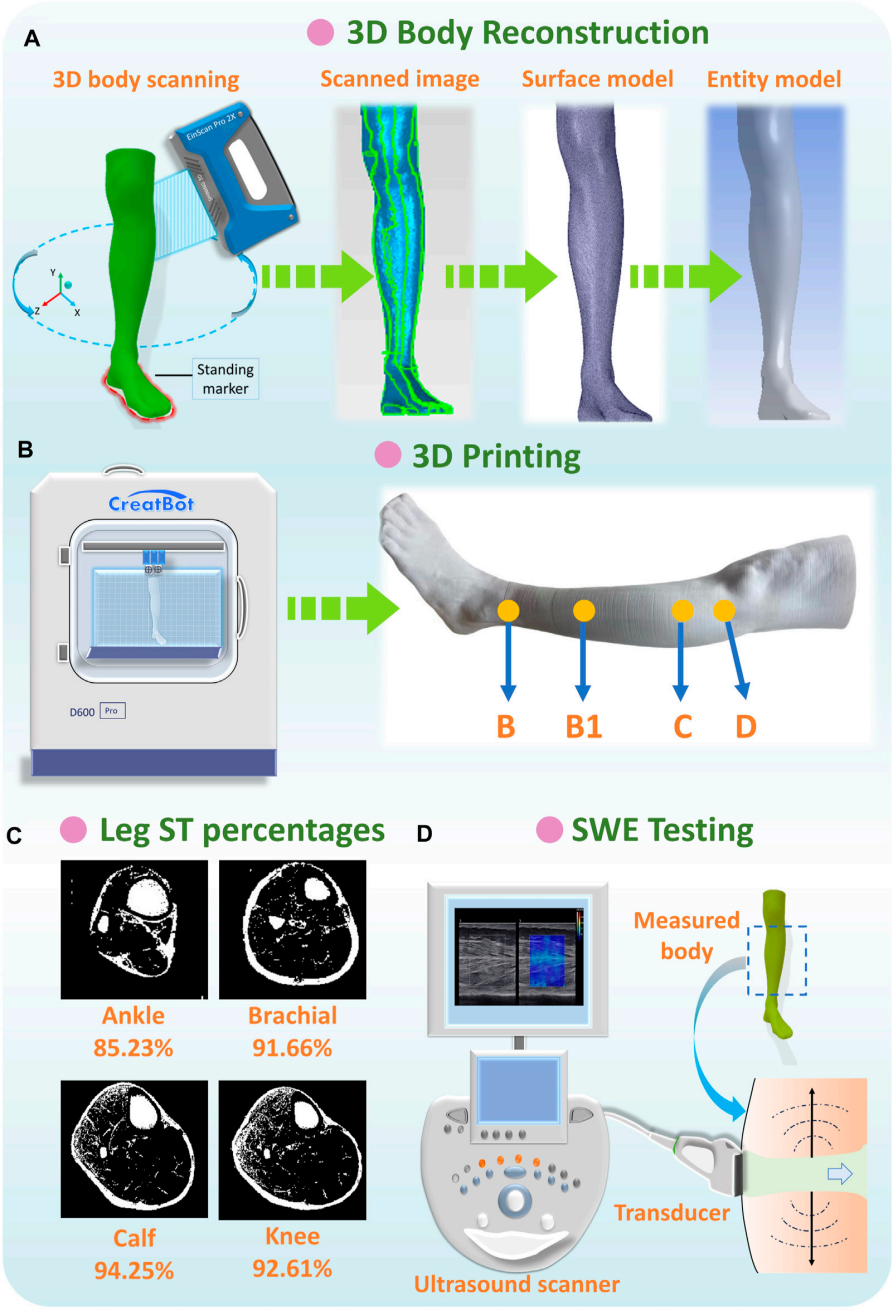
**(A)** Anthropometric data acquisition and reconstruction of lower limbs, **(B)** 3D printing of rigid PLA-based leg models, **(C)** ST percentages of each leg position, and **(D)** SWE testing for lower extremity.

To obtain the lower body surface images for further model reconstruction and 3D printing manufacturing, the handheld professional EinScan-Pro 2X PLUS 3D Scanner (Shining 3D Tech. Co., Ltd. Hangzhou, China) and Solid Edge Shining 3D Edition software were applied with high scan accuracy (scan precision was 0.04 mm) and efficiency (scan speed was 1,500,000 dots/s) (Amornvit and Sanohkan, 2019). Based on the LED light source, participants were requested to stand steadily with torso separation at the instructed boundary pattern markers (Figure 4A). Then, by stably moving and operating the scanner 360° around each subject, the entire lower body surface was produced in cloud points for data acquisition. After body capturing, the initial scanned files were saved in a stereolithography (STL) format and preliminarily processed using the Geomagic Studio 2014 (64 bit) software (Raindrop Geomagic, Research Triangle Park, NC, USA) for the elimination of irrelevant extra scan points, reduction of noises, repair of model holes, and orientation fixation. Then, the 3D entity models were reconstructed by using reverse engineering technology through the SpaceClaim Direct Modeler (SCDM; ANSYS, Pennsylvania, Pittsburgh, USA) and CAD (computer-aided design) systems (Shuxian et al., 2005; Zhan et al., 2011).

After entity model reconstruction, as shown in Figure 4B, rigid leg models were fabricated by the advanced large scale of FDM (fused deposition modeling) Creatbot D600 Pro 3D printer (build volume: 600 mm^3^, precision: 0.05 mm). The sustainable filament materials of poly lactic acid (PLA) are commonly utilized as raw materials in bioprinting, biomedical, tissue engineering, and smart textile industries, *etc*. (Tümer and Erbil, 2021; Plesec et al., 2023). Thus, PLA filaments were applied as the printing materials for replacing the rigid wood lower limb mannequins’ *in vitro* compression measurements due to PLA’s excellent mechanical properties (tensile strength: 46.8 MPa; compressive strength: 17.9 MPa; Young’s modulus: 3.0 GPa) (Gregor et al., 2017). By using 3D printing manufacturing, three PLA-based rigid leg models for each subject with various morphological and geometric characteristics were developed for further pressure estimations.

### 2.4 Determination of ST stiffness properties

To independently investigate the impact mechanisms of various ST stiffness values on pressure performances of CTs, the ST mechanical properties of human lower extremities (below the knee) were referred from relevant previous literature works measured by various protocols and subject groups (Dubuis et al., 2012; Dubuis et al., 2013; Frauziols et al., 2013; Han et al., 2021). The muscle compositions were determined as the major studied components accounting for the proportion ranging from approximately 85%–94% (Figure 4C) (Ye et al., 2023). Thus, in this study, the digitalized Young’s modulus (*E*_*s*_) range of leg ST (muscle) stiffness was 0.0014–0.0030 MPa and divided into three levels (LS-1: 0.0014 MPa, LS-2: 0.0022 MPa, and LS-3: 0.0030 MPa) for further comparisons in the FE modeling system.

Moreover, to obtain the exact ST stiffness values of studied subjects, the real-time shear wave elastography (SWE) was performed using Aixplorer^@^ MultiWave ultrasound system (Supersonic Imagine, Aix-en-Provence, France), to provide the quantitative color-coded map (rectangular box: 1 cm × 1.5 cm) of lower limb tissue elasticity on an anatomic standard B-Mode image (Figure 4D). To ensure elasticity mapping with the SWE sequence, the parameters were set as the musculoskeletal preset and tissue tuner at 1,540 m/s with the resolution mode enabled. For each subject, the depth setting was fixed at 2 cm to display the entire muscle during examination (Ternifi et al., 2020). For physical testing, two leg regions (at the ankle and calf) with four directions (anterior, posterior, medial, and lateral) were measured using a SuperLinear™ SL10-2 transducer array (element number: 192, bandwidth: 2–10 MHz). Then, *E*_*s*_ for each subject was determined using the calculated average values (listed in Table 2) through Eq. 1.

$$E_{S}=3\rho_{m}v^{2},$$ (1)

where *ρ*_*m*_ is the muscle density (1,000 kg/m^3^) and *v* is the shear wave velocity range of 0–7.7 m/s (Dubois et al., 2018).

**TABLE 2:** Measured *E*_*S*_ through the SWE testing.

Subject code	Leg position	*E*_*S*_ (MPa)	Mean *E*_*S*_ (MPa)
S1	B	0.001975	0.002040
	C	0.002145	
S2	B	0.002678	0.002976
	C	0.003273	
S3	B	0.003700	0.002750
	C	0.001800	

### 2.5 Construction of 3D FE homogenous CT-leg systems

First, the geometric models of FE-based CTs were constructed according to the specific physical dimensions (*R*_*F*_ and *L*_*F*_; Table 1) of actual fabrics by adopting ANSYS Workbench Design Modeler software (v19.2, ANSYS, Pennsylvania, Pittsburgh, USA). To ensure the CTs could slide along the leg longitudinal direction, the centers of the cross-sectional leg and CT models were coincident to achieve an alignment by adjusting the model positions. The knitted CTs were commonly assumed as the orthotropic elastic materials (Ye and Liu, 2020), and the inputting fabric property parameters of *MD*, *E*_*Fx*_, *E*_*Fy*_, *v*_*F*_, and *G*_*F*_ were based on the experimental material data (Table 1). In the compression analysis, the neo-Hookean model was typically used for mechanical analysis and pressure prediction, and the STs were basically modeled as hyperelastic, incompressible, homogenous, and isotropic materials. Additionally, the constitutive equation is as follows (Eq. 2) (Dubuis et al., 2012; Arnold et al., 2023):

$$A=C_{10}\left(\bar{I_{1}}-3\right)+D_{1}{\left(J-1\right)}^{2},$$ (2)

where *A* denotes the strain energy density, $\bar{I_{1}}$ is the first deviatoric strain variant, and *J* is the Jacobian determinant of the deformation gradient (for the incompressible materials, the *J* value is 1) (Dubuis et al., 2012; Ye et al., 2023). *C*_*10*_ and *D*_*1*_ can be expressed as below under a linear elastic condition, as follows: (Eq. 3)

$$C_{10}=\frac{S}{2},D_{1}=\frac{B}{2},$$ (3)

where *S* and *B* are the shear and bulk moduli values, respectively.

**FIGURE 5:**
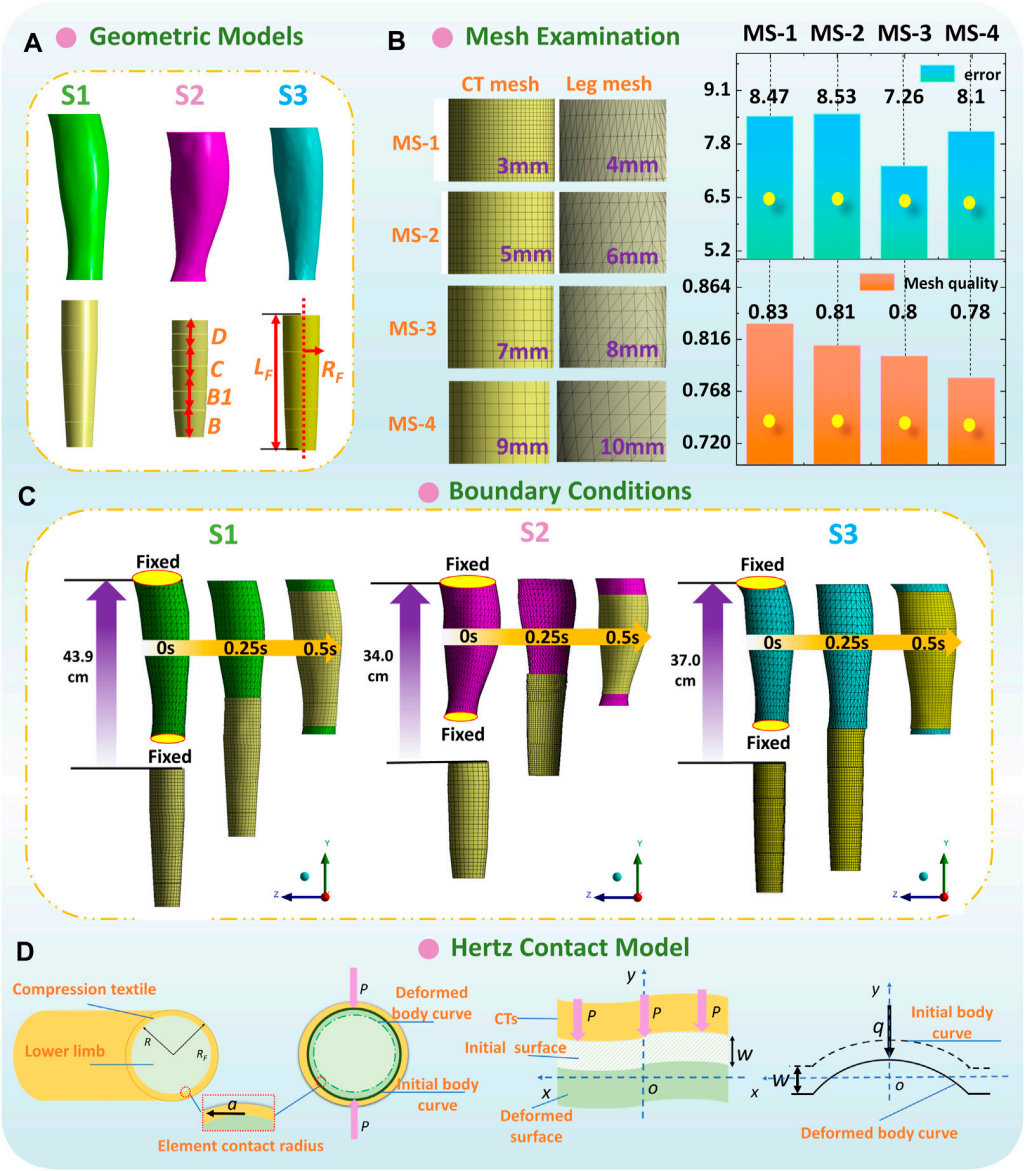
**(A)** Geometric models and **(B)** mesh sensitivity study of 3D FE homogenous modeling. **(C)** Boundary conditions and dynamic wearing process of each FE model. **(D)** Hertz contact model for the CT-leg system.

Thus, for the lower limbs, the mechanical properties of 3D rigid and bio-soft legs were obtained through Young’s modulus of PLA printing material (3 GPa) and biological ST stiffness (S1-0.0034 MPa; S2-0.0050 MPa; S3-0.0046 MPa), respectively. Then, as shown in Figure 5A, to simulate the practical wearing process and analyze the interfacial pressure performances of CTs, the constructed leg models (obtained by section 2.2) and applied CTs of each subject were imported into the Ansys LS-DYNA explicit dynamic solver, to solve the non-linear dynamic equilibrium equation based on the center difference method (Rackauskaite et al., 2017; Liu, 2008) (Eq. (4)).

$$M{\ddot{u}}^{n}=L^{n}-Y^{n}+H^{n},$$ (4)

where *M* is the diagonal mass matrix, ${\ddot{u}}^{n}$ is the nodal acceleration component, *L* is the load, *Y* is the stress component, and *H* is the damped hourglass, where the damped hourglass was applied to reduce hourglass energy in the explicit dynamic model to achieve the simulation accuracy, and *n* represents the *n*th element of a time interval discretization.

Second, as shown in Figure 5B, according to the geometric characteristics of simulated components, CT (shell element) and leg model (solid entity element) were meshed by linear quadrilateral dominant and tetrahedrons elements, respectively. The mesh sensitivity study was performed with various mesh size combinations to examine the mesh independence of proposed FE systems. Through the compared results, 8 mm and 7 mm per element were meshed for CT and leg models, respectively. Thus, the element/node numbers in biomechanical systems for the S1, S2, and S3 subjects were approximately 38500/12300, 60000/14300, and 32000/8000, respectively. The frictional non-linear contact with a coefficient of 0.2 (Huzni et al., 2022) was applied to simulate the interfacial contact conditions.

Third, to promote dynamic wearing and the sliding process, the upper and bottom external surfaces of leg models were fixed to remain stationary and avoid unnecessary movement. Thus, the tubular CTs could slide longitudinally and freely from the distal to the proximal of lower limbs (Figure 5C). The total degrees of freedom (DOF) of built CT-leg models for the S1, S2, and S3 subjects were approximately 34000, 62000, and 45000, respectively. The DOF of each node were six, and conversely, the fixed support condition restricted six DOF. The boundary conditions were applied to the CT geometric models and determined as the longitudinal CT sliding displacements along the *y* axis. The exactly longitudinal sliding displacements of CTs included the distances between the leg bottom to the upper end of CTs, as well as the leg heights (from the B to D positions; S1-35.1 cm, S2-20.1 cm, and S3-21.1 cm) of each subject. Thus, the total longitudinal displacements for boundary conditions (approximately S1-43.9 cm, S2-34.0 cm, and S3-37.0 cm) were determined by not only the distances between the leg bottom to the upper end of CTs but also the leg heights of each subject. Then, the CTs were freely deformed due to the dimensional diversities between CTs and legs along circumferential directions. Interfacial pressures were generated due to the fabric stretching tensions and produced normal forces. Moreover, the dynamic wearing process was 0.5 s, and we captured the pressure mappings at the 1st seconds, when the CTs were stretched steadily along lower bodies and provided constant tensions. Additionally, the simulated pressure magnitudes along the 3D printing rigid legs and soft legs with multiple ST stiffness values were defined as *P*_*LM*_ and *P*_*LS*_, respectively.

### 2.6 Theoretical contact analysis model

To theoretically analyze the impact mechanisms of tissue stiffness on pressure distributions of CTs, the Hertz contact theory (HzT) was used. The HzT model fundamentally leads the basic understanding of the indentation and mechanical interaction between elastic solids (Chen et al., 2017; Zhang et al., 2019), for investigating the geometrical deformation effects on local elastic deformation properties and pressure performances. For CT-leg systems (Figure 5D), the assumptions of HzT are as follows: i) the fabric–tissue contact area can be divided into FE contacts. Each element contact can be analyzed as two camber concave contacts; ii) the radius of contact circle is relatively smaller than those of the elastic fabric and deformed lower extremity; iii) no displacement changes along fabric thickness direction; and iv) during the wearing process, only the normal pressure is generated and transmitted between the frictionless full contact interface.

Through the initial HzT model, the quantitative relationships among the unit pressure magnitude with the body circumferential displacements, the physical dimensions, and mechanical properties of applied legs and CTs are shown as Eq. 5 (Khot and Borah, 2015).

$$\omega =\left(k+k_{F}\right)\frac{\pi q}{4a}\left(2a^{2}-r^{2}\right),$$ (5)

where *w* is the body circumferential displacement (m) and *k* and *k*_*F*_ are the elastic mismatch factors (Pa^−1^) of leg and CT fabric, respectively; *q* is the unit pressure value (Pa); *r* is the point distance from the contact circle surface to the center of the contact circle (defined as zero due to consumption of the full contact condition); and *a* is the radius of the contact circle (m).

The elastic mismatch factors are definitively determined by the mechanical properties of CTs and lower limbs. Thus, *k* and *k*_*F*_ are given by Eq. 6.

$$k=\frac{1-v^{2}}{\pi E_{s}},k_{F}=\frac{1-v_{F}^{2}}{\pi E_{F}},$$ (6)

where *E*_*s*_ and *v* (where *v* is 0.5 (Colombo et al., 2016)) are the tensile elastic Young’s modulus (Pa) and Poisson’s ratios of applied body (along the transverse direction), respectively.

After deformation, as Eq. 7, Hertz also derived *a* and *q* of the contact surface through the determined variables (such as *k* and *R*_*F*_) of the two contacting materials.

$$a={\left[\frac{3\pi F\left(k+k_{F}\right)RR_{F}}{4\left(R+R_{F}\right)}\right]}^{1/3},q=\frac{3F}{2\pi }{\left[\frac{8a^{2}\left(R+R_{F}\right)}{3F\left(k+k_{F}\right)RR_{F}}\right]}^{2/3},$$ (7)

where *F* is the applied normal load of external force (N) and *R* is the radius (m) of the applied leg.

Additionally, through the definition function of interfacial pressure (*P*, Pa), it can be obtained through the applied normal load and contact area (Eq. 8).

$$P=\frac{F}{2\pi a^{2}}.$$ (8)

Therefore, based on the aforementioned HzT model and FE simulated leg circumferential displacement of *w*, the pressure ratios (Δ*P*) between the rigid leg models and soft legs with various stiffness properties can be compared quantitatively (Eq. 9).

$$\Delta P=\frac{P_{LM}}{P_{LS}}=\frac{w_{r}}{w_{s}}\times \frac{\left(k_{s}+k_{F}\right)}{\left(k_{r}+k_{F}\right)}=\frac{w_{ratio}}{k_{ratio}},$$ (9)

where *w*_*r*_ and *w*_*s*_, *k*_*r*_ and *k*_*s*_ are the body circumferential displacements and elastic mismatch factors along the rigid and soft legs, respectively; and *w*_*ratio*_ and *k*_*ratio*_ are the circumferential displacement and elastic mismatch ratios compared between the rigid and soft legs, respectively.

### 2.7 Acceptability analysis of FE modelling and experimental validation study

The acceptability of 3D FE homogenous leg models was compared through the simulated *P*_*LM*_ and experimentally measured *P*_*rigid*_ data obtained along the constructed FE-based and printed PLA leg models with identical mechanical properties (3.0 GPa) and controlled leg morphologies. The pressure prediction errors were compared by the deviation ratio (*DRO*, %; Eq. 10).

$$DRO=\frac{\left|P_{rigid}-P_{LM}\right|}{P_{rigid}}\times 100\%.$$ (10)

To validate the accuracy and applicability of this study, the pressure performances obtained along the subject-specific FE CT-leg system, user-oriented 3D printing rigid model, and biological leg were compared simultaneously. Additionally, the interfacial compression performance experimental evaluations were measured by utilizing the PicoPress^@^ (Microlab Elettronica, Italy) pressure tester (measurement range: 0–189 mmHg and precision: ±3 mmHg). The pressure sensor probe detected the values over the circular area of the four studied leg positions (B, B1, C, and D).

### 2.8 Data analysis

Data analysis was performed using the Statistical Package for the Social Sciences (SPSS) software (version 23.0, IBM Corporation, USA). In this study, four (B, B1, C, and D) positions of three legs and two compression levels (class I and class III) were studied in (i) acceptability estimation of the FE CT-leg system, (ii) comparison study, and (iii) experimental validation study. Thus, the processed sample sizes were as follows: (i) simulated group: 24 and tested group: 24; (ii) comparison study: class I-48 and class III-48 in each compared group; and (iii) the experimental validation data were 24 in each compared group. Based on the processed sample size (*n* < 50), the data normal distribution examinations were objectively conducted by the Shapiro–Wilk statistic (Razali and Wah, 2011). Then, the correlations and significant differences between each variable were tested by performing the Pearson correlation analysis and paired t test, respectively (Bolboaca and jantschi, 2006; Tang et al., 2013). The post hoc test of the Bonferroni correction would be applied when significant differences are identified by the paired t tests. The level of significance was set at *α* = 0.05.

## 3 Results

### 3.1 Acceptability of 3D FE homogenous CT-leg system

**FIGURE 6:**
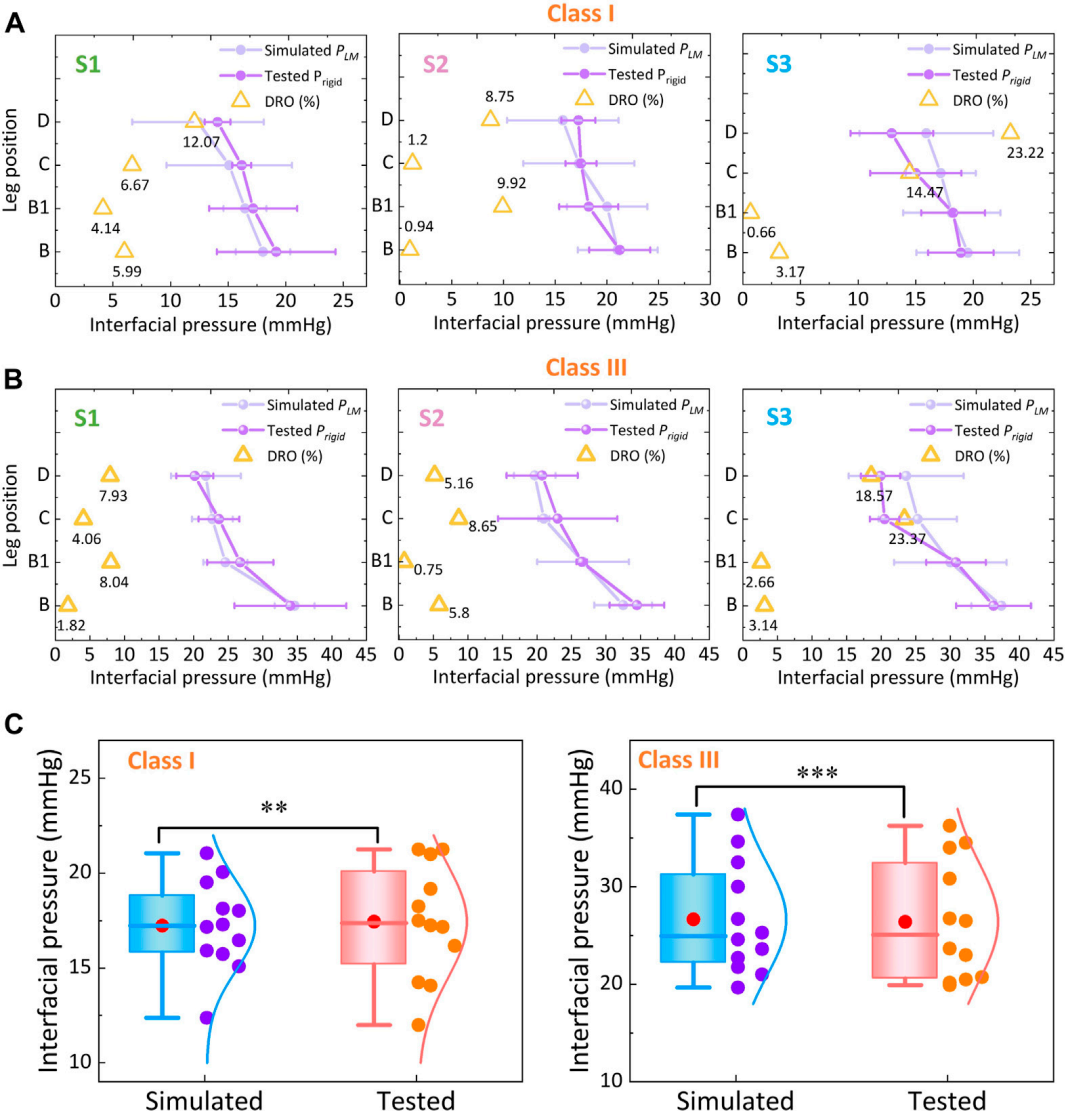
Pressure comparisons between the FE simulated and experimental measured values for **(A)** classes I and **(B)** III CTs. **(C)** Data distribution and correlation analysis of FE-based and tested pressure values (‘*’ represents the *p*-value through the correlation test; **: *Sig.* < 0.01 (correlation); ***: *Sig.* <0.005 (significant correlation)).

Figures 6A, B show the simulated subject-specific *P*_*LM*_ and measured *P*_*rigid*_ values supplied by CTs and corresponding calculated *DROs* of each compression level. For instance, for the ankle (B) position of class I CTs, the simulated values for subjects S1, S2, and S3 were 18.02 ± 2.36 mmHg, 21.05 ± 3.87 mmHg, and 19.52 ± 4.46 mmHg, respectively. The corresponding tested pressure values were 19.17 ± 5.15 mmHg, 21.25 ± 2.95 mmHg, and 18.92 ± 2.84 mmHg, respectively. For the knee (D) position of class III CTs, the simulated values for subjects S1, S2, and S3 were 21.77 ± 5.05 mmHg, 19.68 ± 3.01 mmHg, and 23.62 ± 8.31 mmHg, respectively. The corresponding tested pressure values were 20.17 ± 2.68 mmHg, 20.75 ± 5.16 mmHg, and 19.92 ± 2.84 mmHg, respectively. The degressive pressure gradients indicated the pressure distributions generated by CTs were degressively from the distal to the proximal regions (Shi et al., 2024). Through the applied elastic compression fabrics with various physical circumferential dimensions and mechanical tensile properties, the light (class I) and strong (class III) levels with indicated ranges and standardized degressive pressure gradients were generated along the lower extremities. Specifically, the *P*_*LM*_ values generated by class I tubular fabrics at the ankle (B) positions of each subject were 18.02 ± 2.36 mmHg, 21.05 ± 3.87 mmHg, and 19.52 ± 4.46 mmHg, respectively. For compression class III, the *P*_*LM*_ values were 34.00 ± 8.09 mmHg, 34.50 ± 3.96 mmHg, and 37.39 ± 4.35 mmHg, respectively. According to the comparison tested data of *P*_*rigid*_, the mean *DRO* of constructed FE modeling was approximately 7.45%. Additionally, based on the Shapiro–Wilk statistic results and normal data distribution curves (Figure 6C), the data conformed to the normal distribution. Through the *DRO* values and correlation results, the simulated *P*_*LM*_ of class I (Sig. <0.05; *ρ* = 0.73) and class III (Sig. <0.05; *ρ* = 0.93) CTs were compared as the reasonable agreements with the tested evaluation *P*_*rigid*_ data, respectively (Huang et al., 2015; Shahzad et al., 2015). These results indicated that the established 3D FE homogenous CT-leg systems could be applied for our further explorations with high acceptability and simulation accuracy.

### 3.2 FE comparison among various leg tissue stiffness values

**FIGURE 7:**
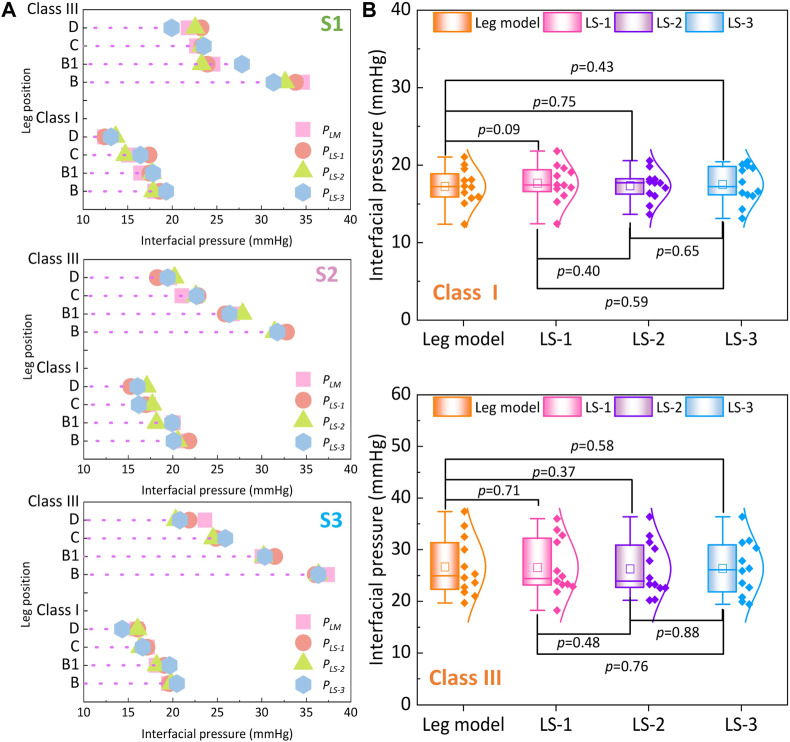
**(A)** Interfacial pressure values generated by different legs with various ST stiffness properties. **(B)** Comparisons of pressure performances generated by class I and class III CTs between each leg stiffness group (the mechanical material stiffness were as follows, leg model: 3.0 GPa, LS-1: 0.0014 MPa, LS-2: 0.0022 MPa, and LS-3: 0.0030 MPa).

To qualitatively investigate the pressure generations along lower bodies with various lower limb tissue characteristics, Figure 7A shows the average compression generations along the simulated rigid (*P*_*LM*_) and soft legs (*P*_*LS*_) with different ST stiffness values supplied by CTs. For the mechanical material, stiffness of the rigid leg model and each tissue group are as follows: leg model: 3.0 GPa, LS-1: 0.0014 MPa, LS-2: 0.0022 MPa, and LS-3: 0.0030 MPa. Through the Shapiro–Wilk statistic tests, the data conformed to the normal distribution. By varying the inputted leg mechanical properties, the exerted interfacial pressure profiles were slightly varied in proposed 3D FE homogenous systems. Furthermore, according to the results of paired t tests (Figure 7B), pressure performances showed no significant differences among each compared leg tissue stiffness group under the light (Sig. >0.05) and strong (Sig. >0.05) external compression levels. It can be demonstrated that the compression performances of CTs with each pressure delivery level have no correlations with tissue characteristics of soft human bio-bodies as well as the applied rigid leg mannequins.

### 3.3 Parametric variations through theoretical HzT model

**FIGURE 8:**
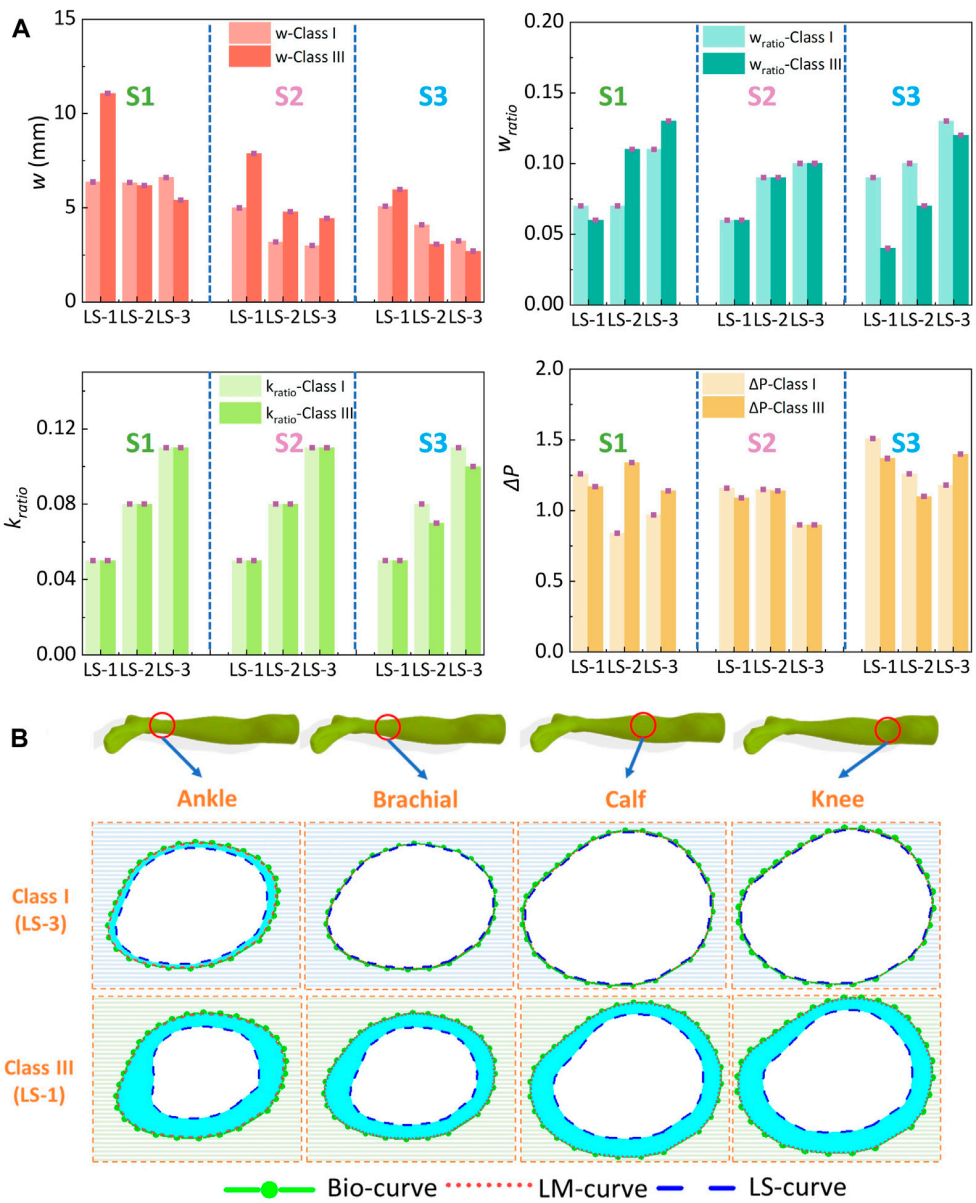
**(A)** Exported leg circumferential curve displacements and calculated parameters through the HzT model of class I and class III CTs. **(B)** Plotted body curves with diverse ST tissue properties and external compressions (from subject S1) (Bio, LM, and LS curves indicated the leg circumferential deformed curves from the subject biological body, 3D rigid PLA model, and simulated soft leg models with ST stiffness values of LS-1 or LS-3).

Based on the HzT model, the pressure differences (ΔP) were determined by the relationships of the leg circumferential displacement (*w*_*ratio*_) and tissue elastic factor (*k*_*ratio*_) ratios. Through the constructed homogenous FE systems with various compression generations and ST stiffness properties, the exported leg circumferential displacements and calculated parameters derived by the HzT model are shown in Figure 8A. For the variables of *w*, it increased with the pressure distribution levels (Sig.<0.05; *ρ* = 0.46), and conversely, decreased with the ST stiffness characteristics (Sig.<0.05; *ρ* = −0.53). For example, Figure 8B shows the compared images plotted by the original bio-body curves, and deformed body curves generated along simulated rigid and soft legs, respectively. The mean *w* value under the light compression level (class I) and maximum ST tissue magnitude (LS-3) was approximately 3.61 ± 0.26 mm. By contrast, under the class III and minimum ST tissue magnitude (LS-1), the *w* value was approximately 11.06 ± 1.36 mm. Based on the *w* variations, the compressed lower limb cross-sectional circumferences varied from 2.27 ± 0.14 cm to 6.95 ± 0.74 cm. Thus, the leg circumferential displacements had correlations with the external pressure levels of CTs and body ST stiffness.

Furthermore, through the calculated parameters of *w*_*ratio*_ and *k*_*ratio*_, the pressure diversities of Δ*P* between the simulated rigid (*P*_*LM*_) and soft (*P*_*LS*_) legs were also obtained quantitatively. ST stiffness properties showed significant positive correlations with deformed variables of *w*_*ratio*_ (Sig.<0.05; *ρ* = 0.64) and tissue-related defined variables of *k*_*ratio*_ (Sig.<0.05; *ρ* = 0.77), but no correlation with the Δ*P* (Sig.>0.05; *ρ* = −0.19). These results indicated that although the external tension forces generated by CTs positively affected the lower body circumferential displacements, the compressed deformed geometric variations caused by diverse tissue stiffness did not lead to proportional changes in pressure performance diversity.

### 3.4 Experimental validation study

**FIGURE 9:**
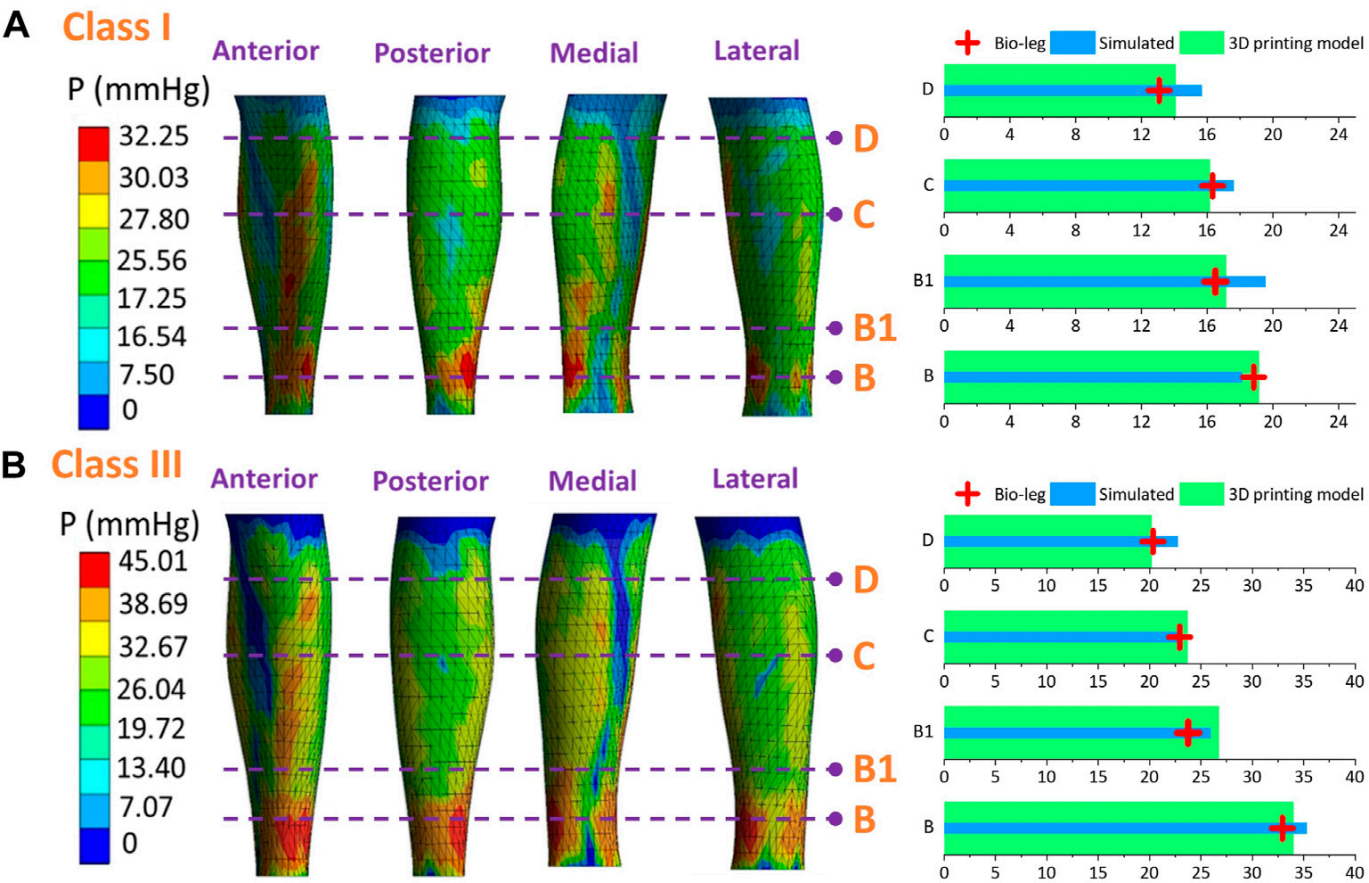
Pressure mappings and experimental validation results of compression classes **(A)** I and **(B)** III for subject S1.

**FIGURE 10:**
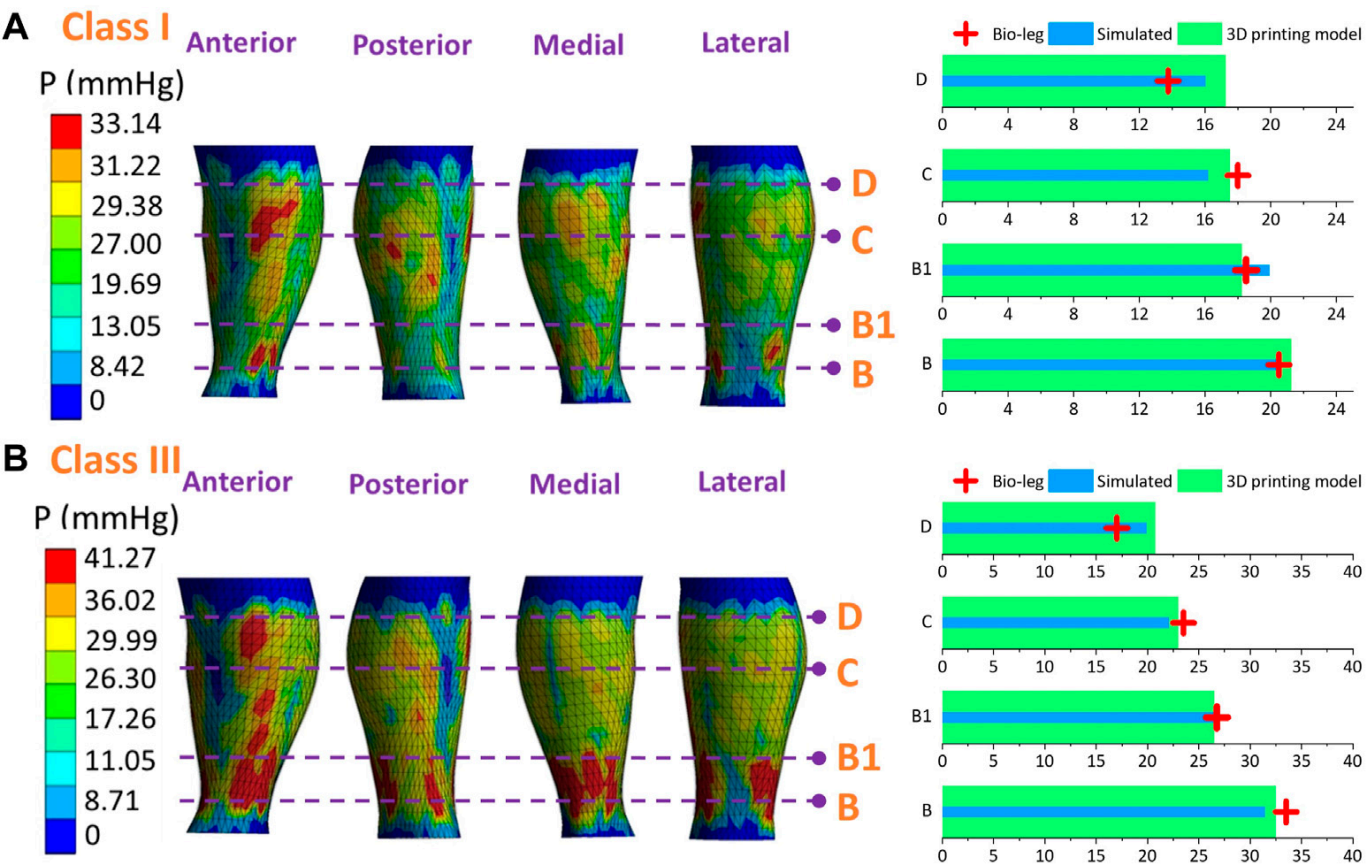
Pressure mappings and experimental validation results of compression classes **(A)** I and **(B)** III for subject S2.

**FIGURE 11:**
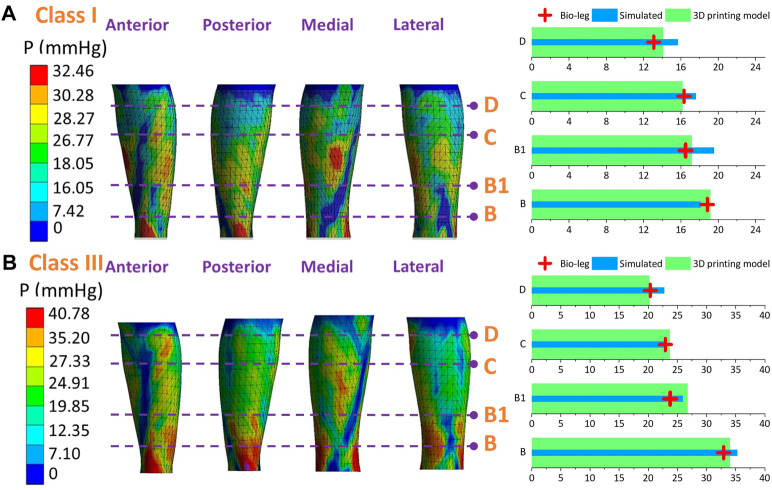
Pressure mappings and experimental validation results of compression classes **(A)** I and **(B)** III for subject S3.

Figures 9–11 represent the subject-specific pressure visualized mappings with class I and class III compression levels delivered by CTs. The inputting leg mechanical properties in FE CT-leg systems were derived from the SWE testing data of each biological body. The standardized pressure gradients and distributions were exerted along each lower extremity and showed individual profile features relating to their morphological characteristics. In addition, the pressure performances along the simulated FE systems (*P*_*LS*_), biological bodies (*P*_*bio*_), and 3D printing models (*P*_*rigid*_) were compared by performing statistical tests. The pressure performances of CTs distributed along biological legs have significant correlations with the values obtained by the simulated FE systems (Sig.<0.05; *ρ* = 0.97) and experimental measurements along the printing leg mannequins (Sig.<0.05; *ρ* = 0.96). Therefore, the proposed 3D FE homogenous CT-leg systems could in practice replace the bio-legs for efficiency pressure prediction simulations. In addition, the non-significant correlations between the ST tissue characteristics and pressure diversities are also validated by experimental tests.

## 4 Discussion

In relevant existing FE CT-leg systems, the lower limbs were reconstructed through medical captured images (i.e., magnetic resonance imaging or computed tomography scanning) and each leg slice was processed individually for composition segmentation and tissue characterization. The mechanical properties of each part of leg were also determined exactly through regional biomaterial measurement. After physical data acquisition and mechanical FE modeling construction, their simulated pressure errors were approximately 5.9%–21.4% (Dai et al., 2007; Dubuis et al., 2013; Ye et al., 2023). For our proposed user-oriented FE models, the lower legs were constructed as simplified homogenous models based on 3D body scanning rather than medical operating equipment with complex physiological structural characteristics. The verification results demonstrate that time-saving and effective CT-leg systems with exact scanned morphological profiles and geometric shapes could facilitate the basic interfacial pressure performance visualization and functional assessment for FE compression studies. Based on the relevant literature works (Kankam et al., 2018; Wang et al., 2018), CTs could increase the venous hemodynamics for improving the clinic therapeutic benefits. The accurate pressure prediction could facilitate the medical efficacy and precision of compression therapy in practical use. Thus, the biomaterial characteristics of the studied main muscle compositions could represent leg mechanical properties in compression simulation to achieve efficient pressure prediction and parameter optimization for the development of CTs.

In previous related studies (Korff et al., 2009; Bosnic et al., 2022), ST stiffness varied during the muscle activation states. For instance, muscular contraction and power production could cause around 150% greater increase in stiffness due to the ankle dorsiflexion in the moving process. For the patient groups with health conditions, ST stiffness caused by progressive muscular diseases also increased by approximately 120% compared with the control groups (Lin et al., 2021). Based on these ST mechanical variations, the *E*_*S*_ of biological legs could approach maximum 0.0045 MPa in dynamic muscle activities, which is still far smaller than the studied rigid leg model (3.0 GPa). Therefore, the main findings for CTs in our study can be applied not only for different bio-bodies with individual tissue diversities but also in various practical wearing and motion scenarios. Through the obtained pressure mappings of simulated results, the insufficient and peak focal pressures were distributed unevenly along each lower extremity. Thus, the biomechanical system could also provide the design strategy (Fontanella et al., 2021) for user-oriented therapeutic CTs with enhanced medical functions and wearing comforts.

Similarly, based on the HzT model, the pressure differences were determined by the relationships of the leg circumferential displacements and tissue elastic factor ratios. The circumferential displacements were varied caused by the external compression levels. In practical application, through the commercial recommended size selection tables (Reich et al., 2016; Liu et al., 2018a), the circumferential dimension ranges between each size of CTs also commonly span around 2 cm–7 cm for identical compression levels, which are consistent with the previously calculated variations of circumference values (2.27 cm–6.95 cm). For elastic CTs, the pressure performances (*P*) are mechanically determined by the fabric tensions (*T*) and body girths (*C*) through Laplace’s law (*P* = *T*/*C*) (Aghajani et al., 2011; Shi et al., 2023). Therefore, through the prescribed pressure ranges of 18–46 mmHg and required CT stretched ratios (15%–80%) (Shi et al., 2023), the garment-based transverse tensile stresses (0.05–0.80 MPa) were relatively smaller to generate the adequate deformed indentations along various biological bodies with diverse tissue properties. Therefore, for the design and development of compression textile-based therapeutic stockings, the pressure generations have no correlations not only with body tissue characteristics but also with the leg mannequin material selections in compression experimental estimations. For the limitation of our study, the mechanical properties of CT were simplified as linear elastic materials under the balanced conditions (CT were stretched steadily and provided constant tensions during the wearing states), and the mechanical behavior variations of CT material time-dependent properties caused by the long-term wearing process would be investigated in our future work.

## 5 Conclusion

This study systematically investigated the biomechanical influences of tissue properties on pressure performances of CTs through FE modeling, theoretical contact modeling, and experimental study. The proposed simplified FE 3D homogenous CT-leg systems effectively and accurately simulated the interfacial pressure performances, and pressure performances of CTs showed no statistically significant differences with the applied lower limbs or leg models with various mechanical tissue properties. The leg circumferential displacements were positively increased by the external fabric tension forces. However, these deformed variations caused by varied biomaterial stiffness could not lead to regular changes in pressure distributions.

Thus, the visualized FE homogenous models provide an efficient biomechanical simulation approach for subject-specific pressure prediction and performance evaluation of CTs. The influencing mechanisms of leg tissue properties on pressure generations of textile-based materials also lead the scientific design principles for the development of CTs with pressure fitness in compression therapy. Therefore, the outcomes of the present study provide not only the biodesign strategy for the pressure management of CTs but also accurate analytical approaches for functional pressure assessments for tailoring rehabilitation equipment and monitoring treatment in compression therapy.

## Data Availability

The original contributions presented in the study are included in the article/Supplementary Material; further inquiries can be directed to the corresponding author.

## References

[B1] Affagard J. S. Bensamoun S. F. Feissel P. J. (2014). Development of an inverse approach for the characterization of *in vivo* mechanical properties of the lower limb muscles. J. Biomech. Eng-t. Asme. 136, 111012. 10.1115/1.4028490 25188787

[B2] Aghajani M. Jeddi A. A. Tehran M. A. (2011). Investigating the accuracy of prediction pressure by laplace law in pressure‐garment applications. J. Appl. Polym. Sci. 121, 2699–2704. 10.1002/app.33640

[B3] Amornvit P. Sanohkan S. S. (2019). The accuracy of digital face scans obtained from 3D scanners: an *in vitro* study. Int. J. Environ. Res. Public Health 16, 5061. 10.3390/ijerph16245061 31842255 PMC6950499

[B4] Arnold N. Scott J. Bush T. R. (2023). A review of the characterizations of soft tissues used in human body modeling: scope, limitations, and the path forward. J. Tissue. Viability 32, 286–304. 10.1016/j.jtv.2023.02.003 36878737

[B5] Avril S. Badel P. Dubuis L. Rohan P. Y. Debayle J. Couzan S. (2011). Patient-specific modeling of leg compression in the treatment of venous deficiency. Patient-specific Model. tomorrow’s Med., 217–238. 10.1007/8415_2011_103

[B6] Barhoumi H. Marzougui S. Abdessalem S. B. (2020). Clothing pressure modeling using the modified laplace’s law. Text. Res. J. 38, 134–147. 10.1177/0887302X19880270

[B7] Bolboaca S. D. Jäntschi L. J. (2006). Pearson versus Spearman, Kendall’s tau correlation analysis on structure-activity relationships of biologic active compounds. Leonardo J. Sci. 5, 179–200.

[B8] Bosnic M. Rasoulian A. Brandon S. C. (2022). Investigating the effects of activation state and location on lower limb tissue stiffness. J. Biomech. 135111032, 111032. 10.1016/j.jbiomech.2022.111032 35305512

[B9] Chen D. Ye Z. Pan Z. Zhou Y. Zhang J. (2017). A permeability model for the hydraulic fracture filled with proppant packs under combined effect of compaction and embedment. J. Pet. Sci. Eng. 149, 428–435. 10.1016/j.petrol.2016.10.045

[B10] Cieślak M. Karaszewska A. Gromadzińska E. Śledzińska K. (2016). I-SCAN method for the assessment of pressure exerted by textile products. Fibers. Text. East. Eur. 6, 121–128. 10.5604/12303666.1221746

[B11] Colombo G. Comotti C. Redaelli D. F. Regazzoni D. Rizzi C. Vitali A. (2016). “A method to improve prosthesis leg design based on pressure analysis at the socket-residual limb interface,” in International Design Engineering Technical Conferences and Computers and Information in Engineering Conference, USA, August 25–28, 2024. 10.1115/DETC2016-60131

[B12] Dai X. Q. Liu R. Li Y. Zhang M. Kwok Y. L. (2007). Numerical simulation of skin pressure distribution applied by graduated compression stockings. Comput. Text., 301–309. 10.1007/978-3-540-70658-8_20

[B13] Dubois G. J. Bachasson D. Lacourpaille L. Benveniste O. Hogrel J. Y. (2018). Local texture anisotropy as an estimate of muscle quality in ultrasound imaging. Ultrasound. Med. Biol. 44, 1133–1140. 10.1016/j.ultrasmedbio.2017.12.017 29428167

[B14] Dubuis L. Avril S. Debayle J. Badel P. J. (2012). Identification of the material parameters of soft tissues in the compressed leg. Comput. Method. Biomec. 15, 3–11. 10.1080/10255842.2011.560666 21809938

[B15] Dubuis L. Avril S. Debayle J. Badel P. J. (2012). Patient-specific FE model of the leg under elastic compression. Int. symposium Comput. methods biomechanics Biomed. Eng. 10.1080/10255842.2012.713610 23009418

[B16] Dubuis L. Rohan C. Y. Avril S. Badel P. Debayle J. (2013). Patient-specific computational models: tools for improving the efficiency of medical compression stockings. Comput. Biomechanics Med. Models, Algorithms Implement., 25–37. 10.1007/978-1-4614-6351-1_4

[B17] Fontanella C. G. Arduino A. Toniolo I. Zampieri C. Bortolan L. Carniel E. L. (2021). Computational methods for the investigation of ski boots ergonomics. Sports. Eng. 24, 15. 10.1007/s12283-021-00352-3

[B18] Frauziols F. Badel P. Navarro L. Molimard J. Curt N. Avril S. (2017). Subject-specific computational prediction of the effects of elastic compression in the calf. Biomechanics Living Organs, 523–544. 10.1016/b978-0-12-804009-6.00024-9

[B19] Frauziols F. Rohan P. Y. Badel P. Avril S. Molimard J. Navarro L. (2013). Patient-specific modelling of the calf muscle under elastic compression using magnetic resonance imaging and ultrasound elastography. Comput. Methods. Biomech. Biomed. Engin. 16, 332–333. 10.1080/10255842.2013.815955 23923963

[B20] Ghosh S. Mukhopadhyay A. Sikka M. Nagla K. J. (2008). Pressure mapping and performance of the compression bandage/garment for venous leg ulcer treatment. J. Tissue. Viability. 17, 82–94. 10.1016/j.jtv.2007.09.013 18722314

[B21] Gong J. M. Du J. S. Han D. M. Wang X. Y. Qi S. L. (2020). Reasons for patient non-compliance with compression stockings as a treatment for varicose veins in the lower limbs: a qualitative study. PloS. One. 15, e0231218. 10.1371/journal.pone.0231218 32343695 PMC7188228

[B22] Gregor A. Filová E. Novák M. Kronek J. Chlup H. Buzgo M. (2017). Designing of PLA scaffolds for bone tissue replacement fabricated by ordinary commercial 3D printer. J. Biol. Eng. 11, 31–21. 10.1186/s13036-017-0074-3 29046717 PMC5641988

[B23] Han Y. He J. Lu Y. J. (2021). Sensitivity of the properties of the graduated compression stocking and soft tissues on the lower limb-stocking interfacial pressure using the orthogonal simulation test. Med. Eng. Phys. 95, 84–89. 10.1016/j.medengphy.2021.07.011 34479696

[B24] Heydon R. (2011). Finite element analysis of knee articular cartilage. Ryerson Univ.

[B25] Hobara H. Kimura K. Omuro K. Gomi K. Muraoka T. Sakamoto M. (2010). Differences in lower extremity stiffness between endurance-trained athletes and untrained subjects. J. Sci. Med. Sport. 13, 106–111. 10.1016/j.jsams.2008.08.002 18951842

[B26] Huang X. Sun J. Li J. (2015). Finite element simulation and experimental investigation on the residual stress-related monolithic component deformation. Int. J. Adv. Manuf. Technol. 77, 1035–1041. 10.1007/s00170-014-6533-9

[B27] Huzni S. Oktianda F. Fonna S. Rahiem F. Angriani L. J. (2022). The use of frictional and bonded contact models in finite element analysis for internal fixation of tibia fracture. Frat. Integrità. Strutt. 16, 130–139. 10.3221/igf-esis.61.09

[B28] Kankam H. K. Lim C. S. Fiorentino F. Davies A. H. Gohel M. S. (2018). A summation analysis of compliance and complications of compression hosiery for patients with chronic venous disease or post-thrombotic syndrome. Eur. J. Vasc. Endovasc. 55, 406–416. 10.1016/j.ejvs.2017.11.025 29329662

[B29] Kankariya N. Laing R M. Wilson C. (2021). Textile-based compression therapy in managing chronic oedema: complex interactions. Phlebology 36, 100–113. 10.1177/0268355520947291 32819205

[B30] Kankariya N. J. (2022). Material, structure, and design of textile-based compression devices for managing chronic edema. J. Ind. Text. 52, 152808372211188. 10.1177/15280837221118844

[B31] Khot S. Borah U. (2015). Finite element analysis of pin-on-disc tribology test. Int. J. Sci. Res. 4, 1475–1480.

[B32] Kim Y. Png C. M. Sumpio B. J. DeCarlo C. S. Dua A. (2021). Defining the human and health care costs of chronic venous insufficiency. Seminars Vasc. Surg. 34, 59–64. 10.1053/j.semvascsurg.2021.02.007 33757637

[B33] Korff T. Horne S. L. Cullen S. Blazevich A. (2009). Development of lower limb stiffness and its contribution to maximum vertical jumping power during adolescence. J. Exp. Biol. 212, 3737–3742. 10.1242/jeb.033191 19880736

[B34] Le S. G. Nordez A. Andrade R. Hug F. Freitas S. Gross R. J. (2017). Stiffness mapping of lower leg muscles during passive dorsiflexion. J. Anat. 230, 639–650. 10.1111/joa.12589 28251615 PMC5382595

[B35] Li Q. Sun G. Chen Y. Chen X. Shen Y. Xie H. (2020). Fabricated leg mannequin for the pressure measurement of compression stockings. Text. Res. J. 92, 3500–3510. 10.1177/00405175221083216

[B36] Lin C. W. Tsui P. H. Lu C. H. Hung Y. H. Tsai M. R. Shieh J. (2021). Quantifying lower limb muscle stiffness as ambulation function declines in duchenne muscular dystrophy with acoustic radiation force impulse shear wave elastography. Ultrasound. Med. Biol. 47, 2880–2889. 10.1016/j.ultrasmedbio.2021.06.008 34284931

[B37] Liu R. Guo X. Lao T. T. Little T. (2017). A critical review on compression textiles for compression therapy: textile-based compression interventions for chronic venous insufficiency. Text. Res. J. 87, 1121–1141. 10.1177/0040517516646041

[B38] Liu R. Guo X. Peng Q. Zhang L. Lao T. T. Little T. (2018b). Stratified body shape-driven sizing system via three-dimensional digital anthropometry for compression textiles of lower extremities. Text. Res. J. 88, 2055–2075. 10.1177/0040517517715094

[B39] Liu R. Lao T. T. Little T. J. Wu X. Ke X. (2018a). Can heterogeneous compression textile design reshape skin pressures? A fundamental study. Text. Res. J. 88, 1915–1930. 10.1177/0040517518779254

[B40] Liu R. Lao T. T. Wang S. (2013). Technical knitting and ergonomical design of 3D seamless compression hosiery and pressure performances *in vivo* and *in vitro* . Fiber. Polym. 14, 1391–1399. 10.1007/s12221-013-1391-x

[B41] Liu R. Xu B. Ye C. (2019). “Biodigital design and functional visualization of multi-class personalized compression textiles for ergonomic fit,” in International Conference on Applied Human Factors and Ergonomics, USA, July 24-27, 2024, 488–499. 10.1007/978-3-030-20444-0_51

[B42] Liu Y. (2008). ANSYS and LS-DYNA used for structural analysis. Int. J. Comput. Aided Eng. Technol. 1, 31–44. 10.1504/IJCAET.2008.021254

[B43] Lu Y. Zhang D. Cheng L. Yang Z. Li J. (2021). Evaluating the biomechanical interaction between the medical compression stocking and human calf using a highly anatomical fidelity three-dimensional finite element model. Text. Res. J. 91, 1326–1340. 10.1177/0040517520979743

[B44] Marinopoulos T. Zani L. Li S. Silberschmidt V. V. (2020). Modelling indentation of human lower-limb soft tissue: simulation parameters and their effects. Contin. Mech. Therm. 35, 939–955. 10.1007/s00161-020-00933-w

[B45] Mo F. Li Y. Li J. Zhou S. Yang Z. J. (2022). A three-dimensional finite element foot-ankle model and its personalisation methods analysis. Int. J. Mech. Sci. 219, 107108. 10.1016/j.ijmecsci.2022.107108

[B46] Morse C. I. (2011). Gender differences in the passive stiffness of the human gastrocnemius muscle during stretch. Eur. J. Appl. Physiol. 111, 2149–2154. 10.1007/s00421-011-1845-z 21298445

[B47] Nemati S. Shojaei S. J. (2019). Investigating effect of compression stocks on tissues of legs. J. Tissues Mater. 2, 14–20. 10.22034/JTM.2019.183444.1016

[B48] Ochi M. Kohara K. Tabara Y. Kido T. Uetani E. Ochi N. (2010). Arterial stiffness is associated with low thigh muscle mass in middle-aged to elderly men. Atherosclerosis 212, 327–332. 10.1016/j.atherosclerosis.2010.05.026 20554280

[B49] Parker M. (2016). Identification of the mechanical properties of living skin: an instrumentation and modelling study.

[B50] Plesec V. Humar J. Dobnik D. P. Harih G. J. M. (2023). Numerical analysis of a transtibial prosthesis socket using 3D-printed bio-based PLA. Materials 16, 1985. 10.3390/ma16051985 36903100 PMC10004398

[B51] Rackauskaite E. Kotsovinos P. Rein G. J. (2017). Model parameter sensitivity and benchmarking of the explicit dynamic solver of LS-DYNA for structural analysis in case of fire. Fire Saf. J. 90, 123–138. 10.1016/j.firesaf.2017.03.002

[B52] Ranger B. J. Moerman K. M. Anthony B. W. Herr H. M. J. (2023). Constitutive parameter identification of transtibial residual limb soft tissue using ultrasound indentation and shear wave elastography. J. Mech. Behav. Biomed. 137, 105541. 10.1016/j.jmbbm.2022.105541 36356423

[B53] Razali N. M. Wah Y. B. (2011). Power comparisons of shapiro-wilk, Kolmogorov-smirnov, lilliefors and anderson-darling tests. J. Stat. Model. Anal. 2, 21–33.

[B54] Reich S. Surhoff S. Stücker M. (2016). Pressure profiles of sport compression stockings. J. Dtsch. Dermatol. Ges. 14, 495–506. 10.1111/ddg.12779 27119471

[B55] Rohan P. Y. Badel P. Lun B. Rastel D. Avril S. J. (2015). Prediction of the biomechanical effects of compression therapy on deep veins using finite element modelling. Ann. Biomed. Eng. 43, 314–324. 10.1007/s10439-014-1121-6 25224080

[B56] Sangpradit K. Liu H. Dasgupta P. Althoefer K. Seneviratne L. (2011). Finite-element modeling of soft tissue rolling indentation. IEEE Trans. Biomed. Eng. 58, 3319–3327. 10.1109/TBME.2011.2106783 21257372

[B57] Shahzad M. Kamran A. Siddiqui M. Z. Farhan M. (2015). Mechanical characterization and FE modelling of a hyperelastic material. Mat. Res. 18, 918–924. 10.1590/1516-1439.320414

[B58] Shi Y. Liu R. Lv J. (2023). Effects of knitting variables for pressure controlling of tubular compression fabrics. Int. J. Mater. Text. Eng. 17, 90–94.

[B59] Shi Y. Liu R. Wong C. Ye C. Lv J. (2024). Prediction of tensile behavior of compression therapeutic biomedical materials by mesoscale laid-in loop model. Polymer 302, 127094. 10.1016/j.polymer.2024.127094

[B60] Shi Y. Ye C. Y. Liu R. (2023). A novel optimization approach for bio-design of therapeutic compression stockings with pressure fit. Comput. Biol. Med. 168, 107768. 10.1016/j.compbiomed.2023.107768 38056207

[B61] Shuxian Z. Wanhua Z. Bingheng L. J. (2005). 3D reconstruction of the structure of a residual limb for customising the design of a prosthetic socket. Med. Eng. Phys. 27, 67–74. 10.1016/j.medengphy.2004.08.015 15604007

[B62] Tang K. Fan J. Zhang J. Sarkar M. Kan C. (2013). Effect of softeners and crosslinking conditions on the performance of easy-care cotton fabrics with different weave constructions. Fiber. Polym. 14, 822–831. 10.1007/s12221-013-0822-z

[B63] Ternifi R. Kammoun M. Pouletaut P. Subramaniam M. Hawse J. R. Bensamoun S. (2020). Ultrasound image processing to estimate the structural and functional properties of mouse skeletal muscle. Biomed. Signal Process. Control 56, 101735. 10.1016/j.bspc.2019.101735 33362876 PMC7757085

[B64] Tümer E. H. Erbil H. (2021). Extrusion-based 3D printing applications of PLA composites: a review. Coatings 11, 390. 10.3390/coatings11040390

[B65] Wang T. Liang F. Liu R. Simakov S. Zhang X. Liu H. (2018). “Model-based study on the hemodynamic effects of graduated compression stockings in supine and standing positions,” in 2018 IEEE-EMBS conference on biomedical engineering and Sciences (USA: IECBES), 27–31. 10.1109/IECBES.2018.8626656

[B66] Ye C. Liu R. (2020). Biomechanical prediction of veins and soft tissues beneath compression stockings using fluid-solid interaction model. Int. J. Biomed. Biol. Eng. 14, 285–290.

[B67] Ye C. Liu R. Ying M. T. Liang F. Shi Y. (2023). Characterizing the biomechanical transmission effects of elastic compression stockings on lower limb tissues by using 3D finite element modelling. Mat. Des. 232, 112182. 10.1016/j.matdes.2023.112182

[B68] Yu W. Fan J. Qian X. M. (2004). A soft mannequin for the evaluation of pressure garments on human body. Sen'i. Gakkaishi. 60, 57–64. 10.2115/fiber.60.57

[B69] Zhan X. H. Li X. D. Liu Y. (2011). Research on reconstruction 3d cad data of automotive panel based on reverse engineering. Adv. Mater. Res. 328, 159–162. 10.4028/scientific.net/AMR.328-330.159

[B70] Zhang Z. Yangyang D. Yanwei H. J. M. (2019). Analytical nonlinear response for a rotor with the Hertz Contact and clearance. Mechanics 25, 473–479. 10.5755/j01.mech.25.6.24790

[B71] Ziaja D. Kocełak P. Chudek J. Ziaja K. J. P. (2011). Compliance with compression stockings in patients with chronic venous disorders. Phlebology 26, 353–360. 10.1258/phleb.2010.010086 21810940

